# Pan-Cancer Analysis Reveals Ribonuclease K (RNASEK) as a Potential Prognostic Biomarker in Pancreatic Cancer and a Diagnostic Indicator Across Multiple Human Cancers

**DOI:** 10.7759/cureus.84574

**Published:** 2025-05-21

**Authors:** Nahla Abdalla Hassan Elsheikh, Nidal A A. Abaker, Majdh F Almrshoud, Fadwa A A. Abdalfadil, Mohamed Alfaki

**Affiliations:** 1 Department of Zoology, University of Nyala, Nyala, SDN; 2 Faculty of Veterinary Medicine, University of Alsalam, Al-Nehood, SDN; 3 Central Research Laboratory, King Saud University, Riyadh, SAU; 4 Faculty of Veterinary Medicine, University of Bahri, Khartoum, SDN; 5 Department of Software Engineering, Faculty of Computer Science, Al-Neelain University, Khartoum, SDN

**Keywords:** diagnostic biomarker, paad, pan-cancer, prognostic biomarker, rnasek

## Abstract

Background: Cancer is marked by rapid abnormal cell growth, leading to high mortality. The human Ribonuclease K (RNASEK) gene is involved in various cellular processes, such as viral infection, immune response, and maintaining cellular homeostasis. RNASEK, found in metazoans, contributes to tumor development, but the lack of systemic pan-cancer investigation into the diagnostic and prognostic function of RNASEK, epigenetic regulation, and interaction with the immune cell infiltration remains unclear. This study investigated RNASEK as a potential pan-cancer biomarker.

Methodology: Public databases such as Tumor Immune Estimation Resource (TIMER), Gene Expression Profiling Interactive Analysis (GEPIA), and University of Alabama at Birmingham Cancer Data Analysis Portal (UALCAN) assessed RNASEK expression patterns. Gene Expression Omnibus (GEO) datasets validated these expressions. UALCAN examined RNASEK's expression differences, DNA methylation, and clinical features. TIMER analyzed SNHG8 expression about immune cell infiltration, while prognosis was evaluated through GEPIA, UALCAN, and Kaplan-Meier (KM) Plotter. cBioPortal reviewed the genetic alterations of RNASEK.

Results: Our study revealed a significant upregulation of RNASEK (*P *< 0.05) in six cancers: bladder (BLCA), cholangiocarcinoma (CHOL), esophageal (ESCA), head/neck squamous cell (HNSC), liver (LIHC), and thyroid (THCA). This was accompanied by notable hypomethylation in BLCA, HNSC, LIHC, and Uterine Corpus Endometrial Carcinoma (UCEC), associated with increased RNASEK expression. Significant differences (*P *< 0.05) were noted between stage 1 and stage 3 in ESCA, HNSC, and THCAas well as significant differences (*P *< 0.05) in HNSC between African-American and Asian populations. Additionally, age-related expression differences were significant (*P *< 0.05) in HNSC across young (21-40 years), middle-aged (41-60 years), and older (61-80 years) groups. A weak positive correlation (*P *< 0.05) existed between RNASEK expression and various immune cell infiltrations such as B cells, CD8+ T-cells, CD4+ T-cells, macrophages, neutrophils, and dendritic cells in patients with BLCA, ESCA, HNSC, and LIHC, while THCA presented moderate negative correlations with CD4+ T-cells and neutrophils. Moreover, High RNASEK expression indicated a good prognosis in pancreatic adenocarcinoma (PAAD) (hazard ratio (HR) 0.49, *P* = 0.0007). RNASEK was altered in less than 1% (95 samples out of 10,967 samples) across various tumor types. The highest alteration rates were identified as significant deletions in miscellaneous neuroepithelial tumors, one case out of 31 cases (3.23%), amplifications in sarcoma, four cases out of 255 cases (1.96%), and mutations in endometrial cancer, which is two cases out of 586 (0.34%).

Conclusions: In conclusion, this study’s pan-cancer analysis revealed that RNASEK could be a potential diagnostic biomarker in six cancer types, including BLCA, CHOL, ESCA, HNSC, LIHC, and THCA, and as a prognostic biomarker in PAAD.

## Introduction

The World Health Organization (WHO) estimates that there will be 19.3 million new cancer diagnoses and approximately 10 million cancer deaths globally in 2020. The incidence and mortality of cancer are increasing rapidly annually, and major obstacles affect the quality of life in every country worldwide, with no cure for cancer [1]. Considering the complexity of tumorigenesis and the successful application of cancer biomarkers [2,3], it is important to be curious about any gene of interest and to explore its prognostic value and underlying molecular mechanisms in cancers [4]. Cancer research has developed exponentially during the past decade because of the revolutionary high-throughput sequencing technology and the data it generates [5].

RNAs are key for gene expression and cell functions. Some RNases break down RNA molecules in various biological processes [6]. Several human RNases are involved in pathogenic conditions, such as inflammatory disorders and autoimmune diseases [7], or the inhibition of tumor growth and metastasis [8], Additionally, RNA degradation by ribonucleases is a key process that affects cell growth, apoptosis, and angiogenesis and plays a critical role in the development of human cancers [9].

The ribonuclease K (RNASEK) is located on chromosome 17 and belongs to a highly conserved protein family in metazoans [6, 10], whose normal function appears to center on intracellular transport and endocytosis [11]. It is a small protein with 95-101 amino acid polypeptide chains. The involvement of RNASEK in regulating essential cellular processes, especially those connected to endocytosis, viral entry, and elements of immune signaling, is demonstrated. RNASEK localizes to the endoplasmic reticulum and cell surface, and it contributes to clathrin-mediated endocytosis and viral uptake through interactions with elements like the vacuolar-type H⁺-ATPase proton pump [11,12].

In addition, previous studies have supported the role of RNASEK in carcinogenesis and metastasis [12]. Moreover, the RNASEK gene is transcribed via expressed sequence tag (EST) analysis in the vast majority of normal and cancerous human tissues; 75% of these sequences are present in cancer tissues, and 25% of them are present in normal tissues [6]. This makes RNASEK a potential diagnostic and/or prognostic biomarker in various types of cancers [5]. Adamopoulos et al. reported eight novel alternatively spliced variants of the human RNASEK gene identified in 55 human cancer cell lines via 3′ RACE and next-generation sequencing (NGS) methodologies [5]. These eight novel RNASEK transcripts are widely expressed in different types of human cell lines, including brain tumors, lung adenocarcinomas, leukemia, lymphomas, melanoma, normal pancreas, normal embryonic kidney, ovarian cancer, cervical cancer, prostate cancer, renal cell carcinoma, bladder cancer (BCLA), hepatocellular carcinoma, gastric adenocarcinoma, colon cancer, brain tumors, lung adenocarcinoma, normal pancreas, normal embryonic kidney, and neck and head squamous cell carcinoma [5]. However, despite the progress made in considering the role of RNASEK in human cancers, the possible participation remains unclear and needs to be further elucidated. While previous studies have suggested the role of RANASK in tumor progression and carcinogenesis, comprehensive pan-cancer research on this gene has not been conducted. Therefore, we conducted a pan-cancer analysis to evaluate RNASEK expression levels across various tumor types, aiming to assess its potential role as a diagnostic and prognostic biomarker.

## Materials and methods

 Gene expression profile of RNASEK across cancers

TIMER, also known as the Tumor Immune Estimation Resource (https://cistrome.shinyapps.io/timer/, accessed in June 2024), is a database that enables the analysis of differential gene expression between tumor and normal tissues [13]. Additionally, we used the GEPIA database (Gene Expression Profiling Interactive Analysis) (http://gepia.cancer-pku.cn/, accessed in June 2024), a web-based tool used for exploring RNASEK expression patterns across different cancers and differences among all stages, via TCGA datasets (The Cancer Genome Atlas Program) [14]. UALCAN (University of Alabama at Birmingham Cancer Data Analysis Portal) (UALCAN (uab.edu, accessed in June 2024) is a user-friendly and powerful OMICS tool for cancer transcriptome analysis that includes RNA-seq expression data from the TCGA dataset [15]. Furthermore, we used the UALCAN database to identify the relationships between RNASEK expression and different pathological and clinical parameters, such as cancer stage, race, and age. The expression pattern of RNASEK was evaluated across various cancers using TIMER, GEPIA, and UALCAN databases.

Methylation analysis of RNASEK

The UALCAN database is a website that allows users to evaluate the epigenetic regulation of gene expression by promoter methylation. Thus, we searched UALCAN (UALCAN (uab.edu, accessed in June 2024) to explore RNASEK promoter DNA methylation levels in various cancers and to determine the differences between tumors and normal tissues [15].

Immune cell infiltration analysis

TIMER (https://cistrome.shinyapps.io/timer/, accessed in June 2024) [13] is an analysis network of tumor immune cell infiltration. It was used to analyze the relationship between RNASEK expression and tumor-infiltrating immune cells in tumors across various cancer types. We subsequently evaluated significant (*P *< 0.05) correlations between the abundance of immune cells, such as B cells, CD8+ T-cells, CD4+ T-cells, neutrophils, macrophages, and dendritic cells, and RNASEK expression.

Overall survival of RNASEK

We investigated the overall survival of RNASEK across various human cancers via three databases. The first database is GEPIA (http://gepia.cancer-pku.cn/, accessed in June 2024). GEPIA is a website that can provide customizable functions such as patient survival analysis. The second database we used was UALCAN (UALCAN (uab.edu), which is an interactive website that can be used to explore patient survival rates among different cancers [15]. Finally, we used Kaplan-Meier (KM) plotter (https://kmplot.com/analysis/), an online survival analysis tool used to assess the relationship between the expression of every gene, including mRNAs, miRNAs, proteins, DNA, and the survival outcomes of patients in 3,500+ samples from 21 tumor types [16].

Genetic alteration analysis of RNASEK

To perform a pan-cancer alteration frequency analysis of RNASEK, we used the cBioPortal database (cBioPortal for Cancer Genomics, accessed in June 2024) [17]. It is a web platform that allows for visualization, exploration, and analysis of cancer data. cBioPortal allows us to uncover the genetic alterations of RNASEK in 10,967 tumor samples from 32 studies, specifically the TCGA Pan-Cancer Atlas studies.

Validation analysis

We performed differential expression analysis via the GEO2R tool (https://www.ncbi.nlm.nih.gov/geo/, accessed in June 2024) [18], an interactive web tool that allows researchers to compare two or more groups of samples to identify differentially expressed genes. Differential expression gene profiles were visualized via volcano plots generated via the bioinformatics.com.cn platform (http://www.bioinformatics.com.cn/srplot) [19], which is an online platform that is mainly used for data analysis and visualization. Statistical analyses were performed based on the criteria of |Log2FC| > 1 and adj *P*-value < 0.05 for the identification of the differentially expressed genes. 

## Results

Gene expression profile of RNASEK across various types of cancers

**Figure 1:**
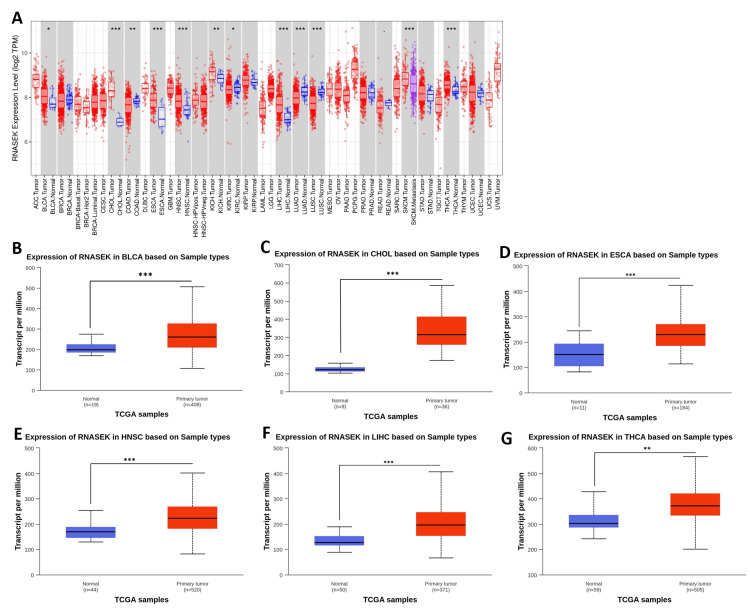
Boxplot showing the mRNA expression levels of RNASEK in normal and cancer tissues via data from the TCGA database. (A) Boxplot from TIMER, the tumor tissues are represented by red dots and boxes, whereas normal tissues are represented by blue dots and boxes. (B-G) Boxplot from UALCAN, the tumor tissues are represented by red boxes, whereas the normal tissues are represented by blue boxes. By cross-referencing, RNASEK was commonly upregulated in six cancers, namely BLCA (B), CHOL (C), ESCA (D), HNSC (E), LIHC (F), and THCA (G). *P ≤ 0.05. **P ≤ 0.01. ***P ≤ 0.001. BLCA, bladder cancer; CHOL, cholangiocarcinoma; ESCA, esophagogastric cancer; HNSC, head and neck squamous cell carcinoma; LIHC, liver hepatocellular carcinoma; THCA, thyroid carcinoma; TIMER, Tumor Immune Estimation Resource; RNASEK, ribonuclease K; TCGA, The Cancer Genome Atlas Program; UALCAN, University of Alabama at Birmingham Cancer Data Analysis Portal

We investigated the differential expression of RNASEK between normal and tumor tissues via the TIMER database (result shown in Figure 1A). RNASEK was significantly upregulated in seven types of cancer (*P* < 0.05), including BLCA, CHOL, ESCA, HNSC, LIHC, KICH, and THCA. However, highly significant (*P *< 0.05) upregulation of skin cutaneous melanoma (SKCM) was observed between the tumor and metastasis groups. Furthermore, there is considerable downregulation in four types of cancer, including colon adenocarcinoma (COAD), kidney renal clear cell carcinoma (KIRC), lung adenocarcinoma (LUAD), and lung squamous cell carcinoma (LUSC). The results from the GEPIA database revealed that RNASEK was significantly upregulated (*P* < 0.05) in cholangiocarcinoma (CHOL) and pancreatic adenocarcinoma (PAAD) (Appendix A). Furthermore, we investigated RNASEK expression via the UALCAN database. The results revealed that RNASEK was significantly (*P* < 0.05) upregulated in 11 types of cancers, including BLCA, CHOL, ESCA, HNSC, LIHC, and THCA (Figures 1B-1G), KICH, KIRP, STAD, THYM, and UCEC (Appendix B). Significant downregulation was detected in three cancers: COAD, LUAD, and LUSC (Appendix B). By cross-referencing the results confirmed by two databases, TIMER and UALCAN, we concluded that RNASEK was commonly upregulated in six types of cancers, including BLCA, CHOL, ESCA, HNSC, LIHC, and THCA (Figures 1A-1G).

RNASEK expression and DNA methylation 

**Figure 2:**
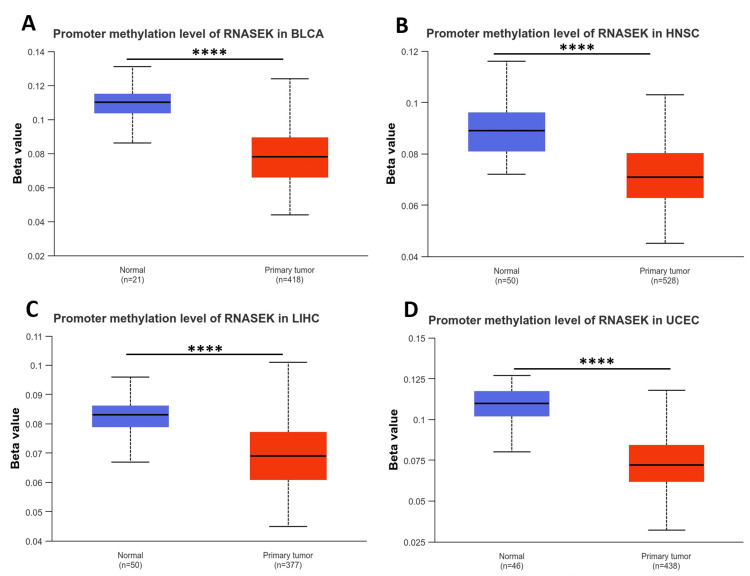
RNASEK promoter methylation analysis using UALCAN via data from the TCGA database. Boxplot illustrates the methylation levels of RNASEK in BLCA (A), HNSC (B), LIHC (C), and UCEC (D) patients. The beta value indicates the level of DNA methylation, ranging from 0 (unmethylated) to 1 (fully methylated). *P < 0.05. **P < 0.01. ***P < 0.001. ****P < 0.0001. BLCA, bladder cancer; HNSC, head and neck squamous cell carcinoma; LIHC, liver hepatocellular carcinoma; THCA, thyroid carcinoma; TIMER, Tumor Immune Estimation Resource; RNASEK, ribonuclease K; TCGA, The Cancer Genome Atlas Program; UALCAN, University of Alabama at Birmingham Cancer Data Analysis Portal; UCEC, uterine corpus endometrial carcinoma

We examined the DNA methylation levels of RNASEK in various tumors using the UALCAN database. The results revealed that the methylation levels of RNASEK in BLCA, HNSC, LIHC, and UCEC tissues were significantly (*P* < 0.05) lower than those in normal tissues, which may explain the high RNASEK expression in these tumors (Figures 2A-2D).

Clinicopathological parameters (stage, race, and age) associated with RNASEK expression

**Figure 3:**
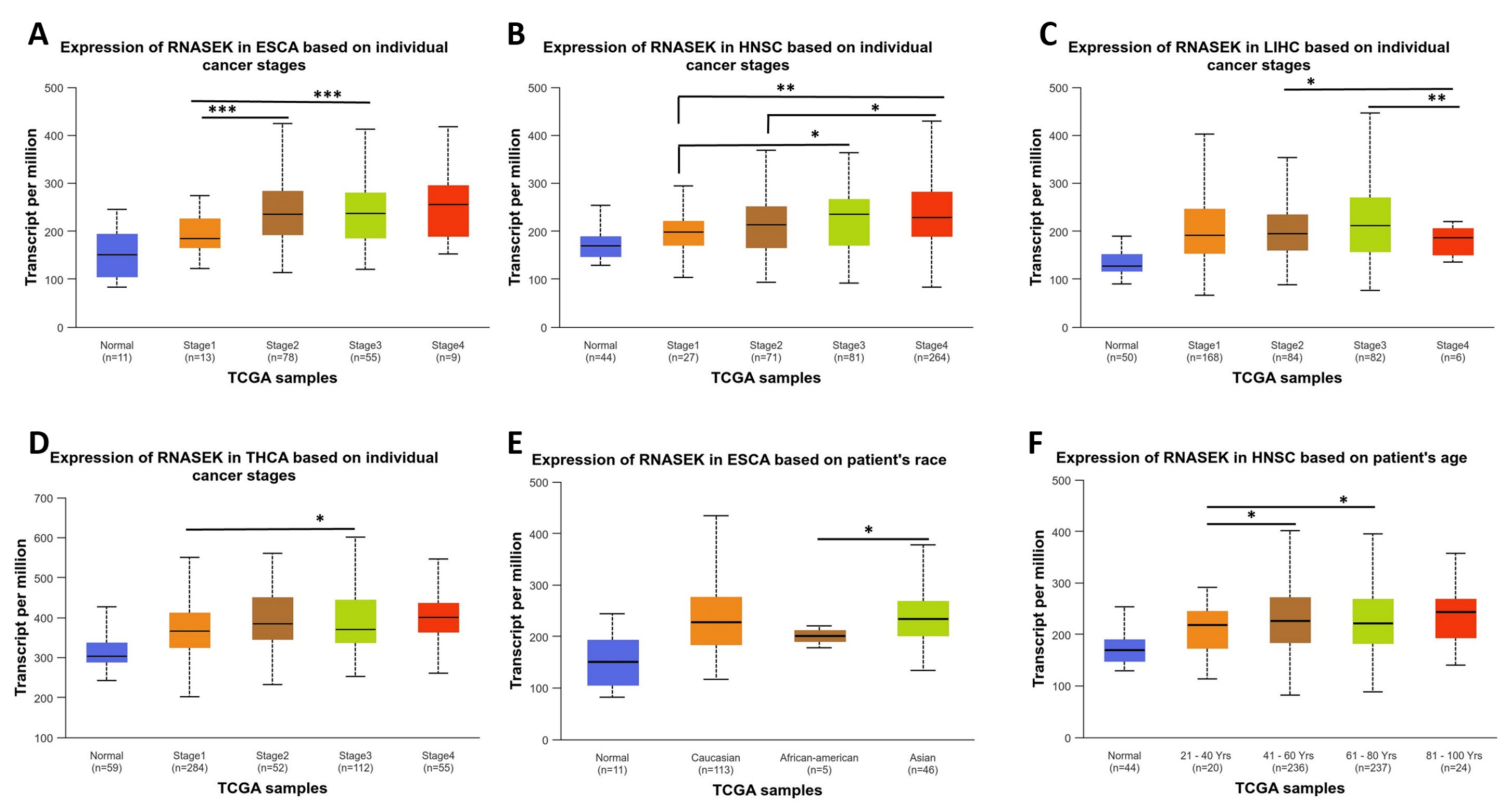
Boxplot from the UALCAN database demonstrating the mRNA expression levels of RNASEK in normal and cancer tissues among stages, race, and age. Cancer stages in (A) ESCA, (B) HNSC, (C) LIHC, (D) THCA, (E) racial differences in ESCA, and (F) age differences in HNSC via data from the TCGA database. *P ≤ 0.05. **P ≤ 0.01. ***P ≤ 0.001. ESCA, esophagogastric cancer; HNSC, head and neck squamous cell carcinoma; LIHC, liver hepatocellular carcinoma; THCA, thyroid carcinoma; RNASEK, ribonuclease K; TCGA, The Cancer Genome Atlas Program; UALCAN, University of Alabama at Birmingham Cancer Data Analysis Portal

We studied RNASEK expression concerning pathological and clinical features, including stage, race, and age, in six types of cancers (BLCA, CHOL, ESCA, HNSC, LIHC, and THCA) via the UALCAN database. Our study observed RNASEK expression variations across cancer stages in ESCA, with significant differences (*P *< 0.001) noted between stages 1 and 2, as well as 1 and 3 (Figure 3A). In HNSC, stage 1 showed marked differences (*P* < 0.05) when compared to stages 3 and 4, and stage 2 differed from stage 4 (Figure 3B). LIHC patients exhibited significant differences (*P* < 0.05) between stages 2 and 3 against stage 4 (Figure 3C). In THCA, stage 1 was significantly different (*P* < 0.05) from stage 3 (Figure 3D). As shown in Figure 3E, racial disparities were evident in ESCA between African-Americans and Asian individuals (*P* < 0.05). Age-wise, we examined the impact of age on RNASEK expression; individuals were categorized into five groups: normal, young (21-40 years), middle-aged (41-60 years), older (61-80 years), and elderly (81-100 years). RNASEK expression significantly varied in HNSC across age groups, which revealed differences (*P* < 0.05) in the young-aged compared to middle-aged and older people (Figure 3F). The comparison between tumor tissues and stages of cancers, race, and age in six cancers under study was provided in Appendix C.

Correlation between RNASEK expression and immune cell infiltration

**Figure 4:**
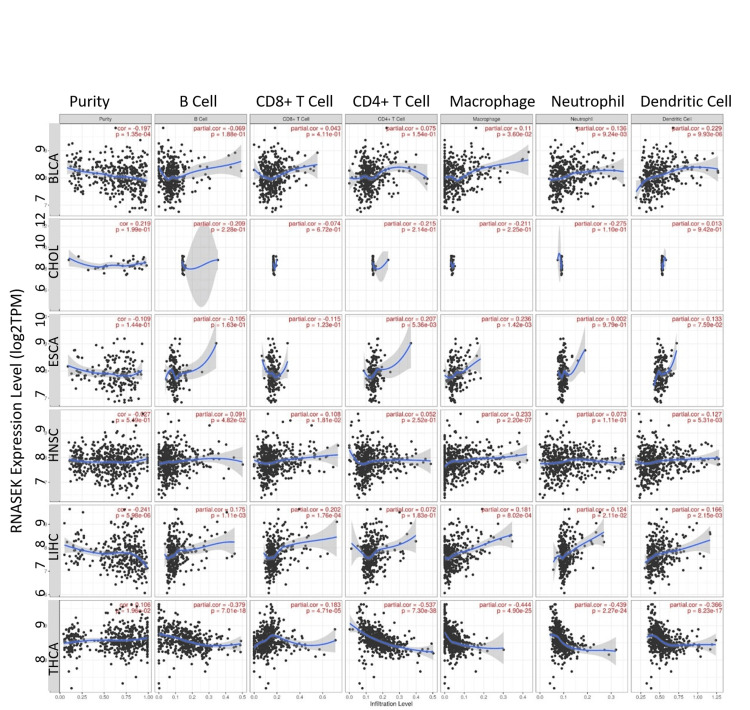
Correlation between RNASEK expression and immune cells infiltration. Scatterplots from TIMER illustrating the correlation of RNASEK expression with immune infiltration levels of B cells, CD8+ T-cells, CD4+ T-cells, macrophages, neutrophils, and dendritic cells in  BLCA, ESCA, HNSC, LIHC, CHOL, and THCA patients. TPM, transcript per million; log2, logarithm(base2); partial cor, partial correlation; *p*, *P*-value; BLCA, bladder cancer; ESCA, esophagogastric cancer; HNSC, head and neck squamous cell carcinoma; LIHC, liver hepatocellular carcinoma; CHOL, cholangiocarcinoma; THCA, thyroid carcinoma; TIMER, Tumor Immune Estimation Resource; RNASEK, ribonuclease K

We investigated the correlation between RNASEK expression and immune cells (B cells, CD8+ T-cells, CD4+ T-cells, macrophages, neutrophils, and dendritic cells) in six cancers, including BLCA, CHOL, ESCA, HNSC, LIHC, and THCA. As shown in Figure 4, our study found no significant correlation in CHOL, while identifying a significant (*P* < 0.05) weak positive correlation between RNASEK expression and macrophages in BLCA (correlation = 0.11, *P* = 0.036), ESCA (correlation = 0.236, *P* = 0.0014), HNSC (correlation = 0.233, *P *= 2.20E-07), and LIHC (correlation = 0.181, *P* = 0.0008). Additionally, we observed weak positive correlations with B cells in HNSC (correlation = 0.1, *P* = 0.048), and LIHC (correlation = 0.175, *P* = 0.0011), and CD8+ T-cells in HNSC (correlation = 0.108, *P* = 0.018), LIHC (correlation = 0.202, *P* = 0.0001), and THCA (correlation = 0.183, *P* = 4.71E-05). CD4+ T-cells had a weak positive correlation with ESCA (correlation = 0.207, *P* = 0.005). Neutrophils showed weak positive correlations in BLCA (correlation = 0.136, *P* = 0.009) and LIHC (correlation = 0.124, *P* = 0.021). Dendritic cells have a weak positive correlation with BLCA (correlation = 0.229, *P* = 9.93E-06), HNSC (correlation = 0.127, *P* = 0.0053), and LIHC (correlation = 0.166, *P* = 0.0021). Notably, THCA exhibited moderate negative correlations with several immune cells, including macrophages (correlation = -0.444, *P* = 4.90E-25), B cells (correlation = -0.379, *P* = 7.01E-18), CD4+ T-cells (correlation = -0.537, *P *= 7.30E-38), neutrophils (correlation = -0.439, *P* = 2.27E-24), and dendritic cells (correlation = -0.366, *P* = 8.23E-17).

Overall survival analysis of RNASEK across all human cancers

**Figure 5:**
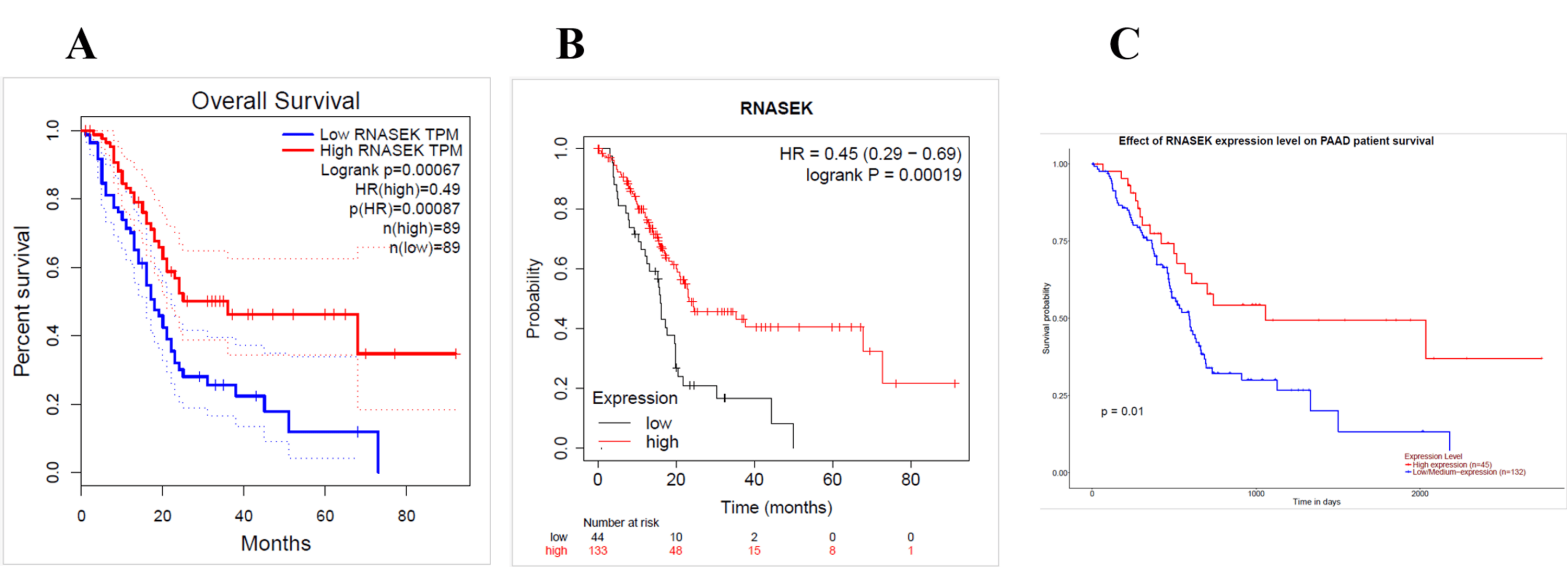
Representative RNASEK survival curves of prognostic analysis obtained via (A) GEPIA, (B) Kaplan-Meier, and (C) UALCAN in PAAD patients. Effects of RNASEK expression on overall survival (*P* < 0.05). HR, hazard ratio, TPM, transcripts per million; p, *P*-value; RNASEK, ribonuclease K; GEPIA, Gene Expression Profiling Interactive Analysis; UALCAN, University of Alabama at Birmingham Cancer Data Analysis Portal; PAAD, pancreatic adenocarcinoma

We identified the relation between RNASEK expression and overall survival by using the GEPIA, UALCAN, and Kaplan-Meier (KM) databases. The overall survival data obtained via GEPIA were statistically analyzed, and the *P*-value was < 0.05; the low and high cutoffs were 50%, and the confidence interval (CI) was 95%. We found that high expression of RNASEK was associated with good prognosis in PAAD patients (hazard ratio (HR): 0.49, *P* = 0.0007) (Figure 5A) and acute myeloid leukemia (LAML) patients (HR: 0.53, *P* = 0.029) (Appendix D). According to the KM database, our results revealed a correlation between high RNASEK expression and good prognosis in five types of cancers: BLCA (HR: 0.57, *P* = 0.002), cervical squamous cell carcinoma (CESC) (HR: 0.58, *P* = 0.021), KIRP (HR: 0.43, *P* = 0.013), ovarian cancer (OV) (HR: 0.64, *P* = 0.005), sarcoma (SARC) (HR: 0.55, *P* = 0.019) (Appendix D) and PAAD (HR: 0.45, *P* = 0.0001) (Figure 5B). In addition, high RNASEK expression was correlated with poor prognosis in HNSC (HR: 1.32 (1-1.74), *P* = 0.048) and KIRC (HR: 2.3 (1.65- 3.21), *P* = 0.048) (Appendix D). Furthermore, overall survival analysis via UALCAN revealed that high expression of RNASEK was associated with good prognosis in three cancers, namely, kidney renal papillary cell carcinoma (KIRP) (*P* = 0.014), OV (*P* = 0.015) (Appendix D), and PAAD (*P* = 0.01) (Figure 5C), whereas poor prognosis was detected in ESCA (*P* = 0.039), KICH (*P* = 0.019), and READ (*P* = 0.029) (Appendix D). By cross-referencing the results obtained from three databases (GEPIA, KM, and UALCAN), we found that PAAD was associated with good prognosis and high expression of RNASEK (Figures 5A-5C).

Genetic alteration analysis of RNASEK

**Figure 6:**
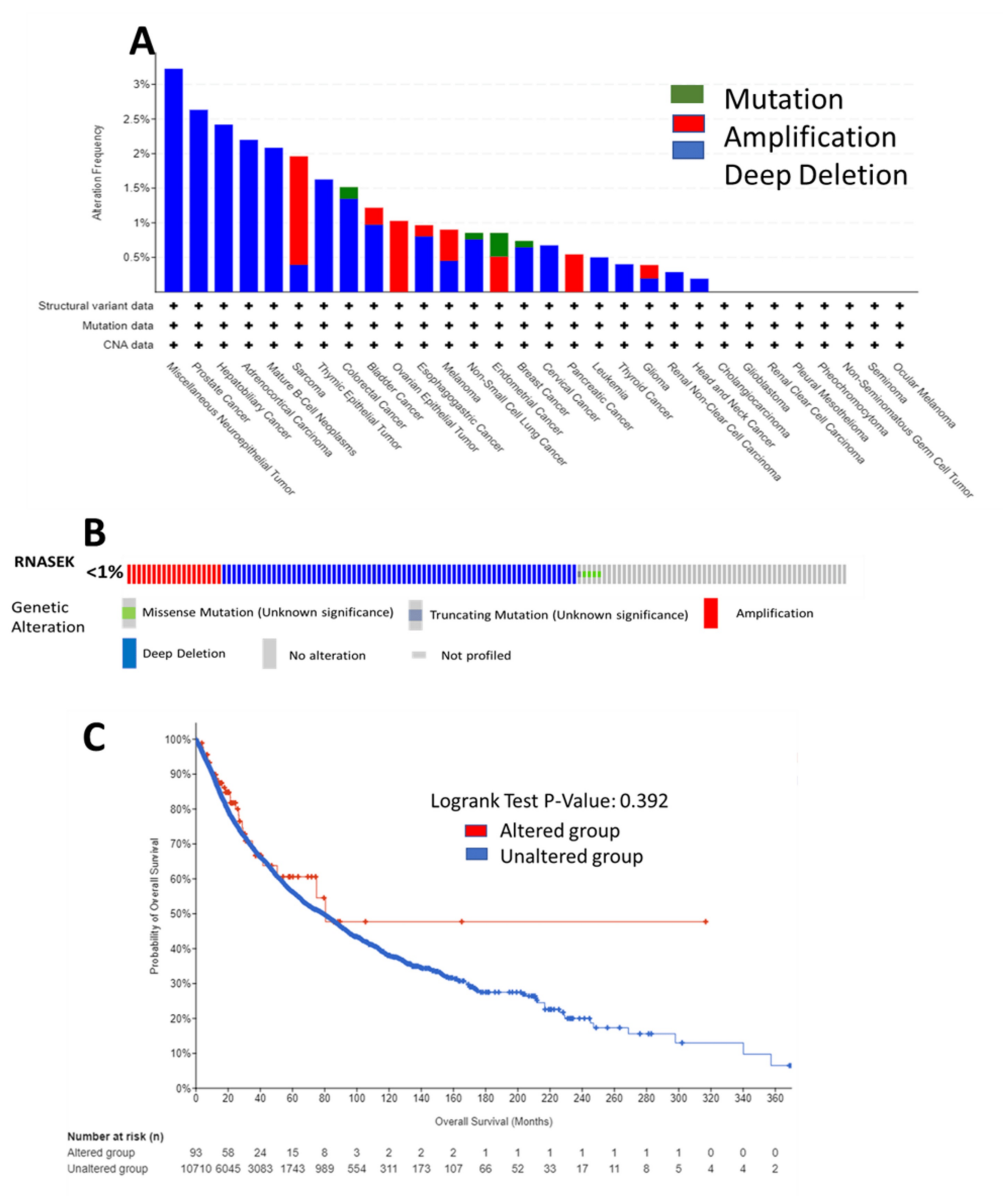
Genetic alteration analysis of RNASEK via the cBioPortal database. (A) Alteration frequency of RNASEK among various cancers. (B) Genetic alterations in RNASEK. (C) Survival rates between altered and unaltered groups. RNASEK, Ribonuclease K

We analyzed RNASEK genetic alterations in the TCGA Pan-Cancer Atlas across 32 studies (10,967 samples) via the cBioPortal database; RNASEK was altered in 95 samples (<1%). As shown in Figure 6A, the RNASEK alterations included deep deletions, followed by amplification and mutation. In the form of deep deletions, RNASEK was altered in nineteen types of cancer, with the highest rate observed in miscellaneous neuroepithelial tumors, where one case out of 31 (3.23%) was affected. Amplification was identified in eight types of cancer, including SARC, BLCA, OV epithelial tumor, esophagogastric cancer (ESCA), melanoma, endometrial cancer, PAAD, and glioma, with the highest rate detected in SARC, at four cases out of 255 (1.96%). The frequency of mutations was found in four types of cancer: endometrial cancer, with two cases out of 586 (0.34%); colorectal cancer, with one case out of 594 cases (0.17%); non-small cell lung cancer, with one case out of 1,053 cases (0.09%); and breast cancer, with one case out of 1,084 cases.

Validation

**Figure 7:**
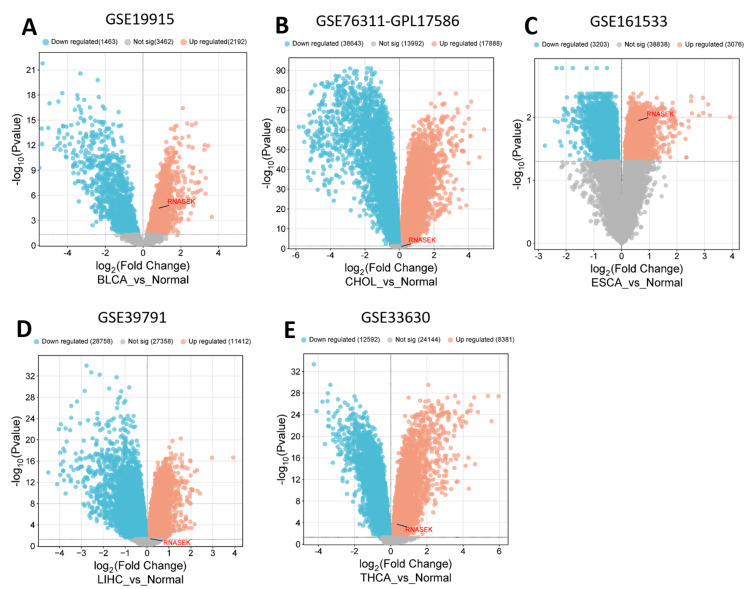
Volcano plots illustrate the differential gene expression of RNASEK in BLCA (A), CHOL (B), ESCA (C), LIHC (D), and THCA (E) via the bioinformatics.com.cn platform. The upregulated genes are depicted as red dots, whereas the downregulated genes are presented as blue dots. The gray marked zone represents the genes that have no significance according to the |Log2FC| > 1 adjusted *P*-value < 0.05. BLCA, bladder cancer; CHOL, cholangiocarcinoma; ESCA, esophagogastric cancer; LIHC, liver hepatocellular carcinoma; THCA, thyroid carcinoma

We validated the expression of RNASEK in five cancers, including BLCA, CHOL, LIHC, ESCA, and THCA, via public datasets obtained from the Gene Expression Omnibus (GEO). We employed the GEO2R tool to indicate the differential expression of RNASEK (|Log2FC| > 0.5, adjusted *P*-value < 0.05), and we utilized the http://www.bioinformatics.com.cn/srplot platform to visualize the volcano plots for differentially expressed genes. As shown in Figure 7, RNASEK expression was significantly upregulated in five cancers: BLCA (GSE19915-GPL3883, tumor = 76 samples, normal = 8 samples), CHOL (GSE76311-GPL17586, tumor = 92 samples, non-tumor = 93 samples), ESCA (GSE161533, tumor = 40 samples, normal = 16 samples), LIHC (GSE139791, tumor = 72 samples, non-tumor = 72 samples), and THCA (GSE133630, tumor = 49 samples, non-tumor = 45 samples).

## Discussion

Researchers worldwide consider pan-cancer biomarker analysis as a useful and cost-effective screening tool to better understand the role of blood or other body fluids as biomarkers [20,21]. This study investigated how RNASEK functions as a diagnostic and prognostic biomarker in a human pan-cancer dataset. Moreover, the expression of the RNASEK gene and its potential implications in various types of cancer have been explored. We used a variety of databases from TCGA, such as TIMER, GEPIA, UALCAN, cBioportal, and KM, for a comprehensive examination of RNASEK in 33 types of tumors, covering gene expression, prognosis, gene alterations, immune infiltration, DNA methylation, and validation.

Our findings demonstrated significant RNASEK upregulation across six cancer types: BLCA, CHOL, ESCA, HNSC, LIHC, and THCA, which aligns with previous research showing threefold higher RNASEK expression in various cancer tissues like heart, brain, placenta, lung, liver, skeletal muscle, kidney, and pancreas compared to normal tissues [6]. RNASEK is expressed as one main transcript in nearly all human tissues and developmental stages, as well as in many carcinomas, which may explain its involvement in the carcinogenesis process [22]. Identifying eight novel alternatively spliced RNASEK variants with widespread expression in human cancer cell lines further emphasizes its biological significance in cancer development [5]. Furthermore, the importance of RNASEK in cancer is supported by the general role of ribonucleases in RNA degradation, which influences critical cellular processes including growth, apoptosis, and angiogenesis [9]. Additionally, other human RNases have been implicated as pathogenic factors in inflammatory disorders and autoimmune diseases [7] and the suppression of tumor growth and metastasis [8]. However, the specific role of human RNASEK in cancer progression remains poorly understood, and further investigations are needed.

Epigenetic modifications, such as DNA methylation can significantly influence gene expression without altering the underlying DNA sequence, making them key targets for cancer research, and recognized as crucial factors in cancer development and progression, regulate cell proliferation, and potential biomarkers for early detection, diagnosis, and treatment of various tumors [23]. Abnormal patterns of DNA methylation have been linked to the occurrence and proliferation of cancer [24]. Our study found that RNASEK expression is elevated in several cancer types (BLCA, HNSC, LIHC, UCEC) due to reduced DNA methylation. This suggests that RNASEK methylation could serve as a potential biomarker for prognosis in these patients.

Our analysis of RNASEK expression across cancer stages revealed significant differences between stage 1 compared to stage 3 in ESCA, HNSC, and THCA, and significant differences were observed between stages 2 and 3 when compared to stage 4 in LIHC patients. These differences among stages suggest RNASEK's fundamental role in these cancers' pathogenesis and potential involvement in advanced disease progression. These findings present an interesting contrast with previous research on prostate cancer, where RNASEK downregulation was associated with cancer development, and its overexpression correlated with reduced tumor aggressiveness and improved survival [25]. 

Our analysis of RNASEK expression across racial groups demonstrated significant differences among HNSC, LIHC, and THCA cancers between normal control tissue and all three major racial groups under study (Caucasian, African American, and Asian populations). ESCA showed variations between African American individuals compared to Asian individuals. This finding could suggest that RNASEK upregulation mechanisms in these cancers may be conserved across different genetic backgrounds. For BLCA and ESCA cancers, significant differences were found between normal tissue and both Caucasian and Asian groups, but not African American populations, potentially indicating protective factors or distinct environmental influences in African American populations for these specific cancers. In CHOL, significant differences were only observed between normal and Caucasian groups, suggesting possible genetic or environmental factors specific to Caucasian populations that influence RNASEK expression in this cancer type. These varied racial patterns in RNASEK expression demonstrate the complex interaction between genetic predisposition, environmental factors, and cancer development, emphasizing the importance of considering racial differences in cancer research and treatment strategies.

Regarding age, differences in RNASEK expression were also found to be an important factor in our research. By categorizing individuals into five age groups (normal, young, middle-aged, older, and elderly), we identified different patterns among various forms of cancer in comparison to normal controls. In CHOL, LIHC, and THCA, all age categories older than 20 showed significant differences. Variations are also observed in the young-aged compared to middle-aged and older people in HNSC. This broad age range of RNASEK upregulation suggests that its role in these cancers may not be strictly age-dependent. However, further investigation into the mechanisms underlying the role of RNASEK in cancer biology as a diagnostic or therapeutic intervention.

Tumor immune infiltrating cells are closely associated with tumor progression, immune checkpoint inhibition function, and patient prognosis [26-28]. Our results revealed a consistent weak positive correlation between RNASEK expression and macrophage abundance in BLCA, ESCA, HNSC, and LIHC patients. This relationship suggests that RNASEK may play a role in macrophage recruitment or survival within the tumor microenvironment. Thus, tumor-associated macrophages play complex roles in cancer pathophysiology. The results for LIHC were particularly noteworthy, as RNASEK expression showed weak positive correlations also with B cells, CD8+ T-cells, neutrophils, and dendritic cells. This association with multiple immune cell types suggests that in LIHC, RNASEK might be involved in tumor progression. The findings in THCA, RNASEK expression showed a weak positive correlation with CD8+ T-cells, while it exhibited significant weak negative correlations with the infiltration of B cells, CD4+ T-cells, macrophages, neutrophils, and dendritic cells. This contrasting pattern suggests a complex and potentially unique role for RNASEK in the immune landscape of thyroid cancer. Our study provides novel insights into the potential role of RNASEK in cancer immunity. However, further research is necessary to fully elucidate its immunological functions and mechanisms of action in human cancers.

In various types of cancer, the mRNA expression of RNASEK showed notable prognostic connections in pan-cancer patients. Elevated RNASEK levels were linked to good prognosis in LAML, BLCA, CESC, KIRP, OV, and PAAD, but poor prognosis in ESCA, HNSC, KICH, KIRP, and READ patients. On the other hand, in late-stage ovarian cancer, increased expression of RNASEK has been associated with resistance to chemotherapy and worse survival rates. The differing observations indicate that the involvement of RNASEK in cancer is intricate and reliant on context, varying based on cancer type and stage of progression [29]. In summary, PAAD was selected as a cancer with great potential prognostic value. However, the impact of RNASEK on survival varied across different cancers, suggesting its complex role in tumorigenesis. Further investigation is required to elucidate the specific mechanisms underlying RNASEK's prognostic value in these cancers. 

Furthermore, our genomic analysis identified frequent genetic alterations in RNASEK particularly deep deletions in miscellaneous neuroepithelial tumors, amplifications in SARC, and finally mutations in endometrial cancers. This finding suggests that RNASEK might play a role in the progression of tumors in these cancers. Another study stated the potential importance of Genetic mutations in tumor development and progression [30].

Limitations

Our study provides valuable insights into the role of RNASEK across different cancers using publicly available databases. However, some limitations should be considered. First, all the analyses were based on bioinformatics tools, further experiments to clarify the precise mechanisms by which RNASEK contributes to cancer development and progression are needed in the future. Second, the original data of these databases were mainly derived from databases, the method of collecting and processing data may not be consistent from database to database, which might cause systematic bias. Finally, the sample size and diversity of patient populations in public databases may not fully represent the full scope of cancer globally, affecting the ability to apply findings to diverse ethnicities and regions. The study primarily focuses on mRNA expression levels of RNASEK. Further examination of protein expression, post-translational modifications, and other functional aspects of the protein is advantageous. 

## Conclusions

In conclusion, this study’s pan-cancer analysis revealed that RNASEK could be a potential diagnostic biomarker in six types of cancers, including BLCA, CHOL, ESCA, HNSC, LIHC, and THCA. Further, we confirmed RNASEK upregulation in BLCA, CHOL, LIHC, ESCA, and THCA by the GEO datasets. Additionally, it shows promise as a prognostic biomarker for PAAD. Our findings highlighted the significant reduction in the methylation levels of RNASEK in BLCA, HNSC, LIHC, and UCEC tissues, which may explain the high RNASEK expression in these tumors. Our study showed marked RNASEK expression variations across cancer stages in ESCA, HNSC, LIHC, and THCA patients. Racial differences were evident in ESCA between African-American and Asian populations. Age-wise, RNASEK expression significantly varied in HNSC across age groups. Furthermore, there is a weak positive correlation between RNASEK expression and macrophages in BLCA, ESCA, HNSC, and LIHC. Weak positive correlations were also noted with B cells and CD8+ T-cells in HNSC, LIHC, and THCA. CD4+ T-cells showed a weak positive correlation with ESCA. Additionally, neutrophils and dendritic cells displayed weak positive correlations in BLCA, HNSC, and LIHC. Conversely, THCA demonstrated moderate negative correlations with various immune cells, including macrophages, B cells, CD4+ T-cells, neutrophils, and dendritic cells. Moreover, PAAD was linked with good prognosis and high expression of RNASEK. RNASEK alterations were rare across tumor types; they exhibited deep deletions in miscellaneous neuroepithelial tumors, amplification in SARC, and mutations were identified in endometrial cancer.

## Figures and Tables

**Figure 8:**
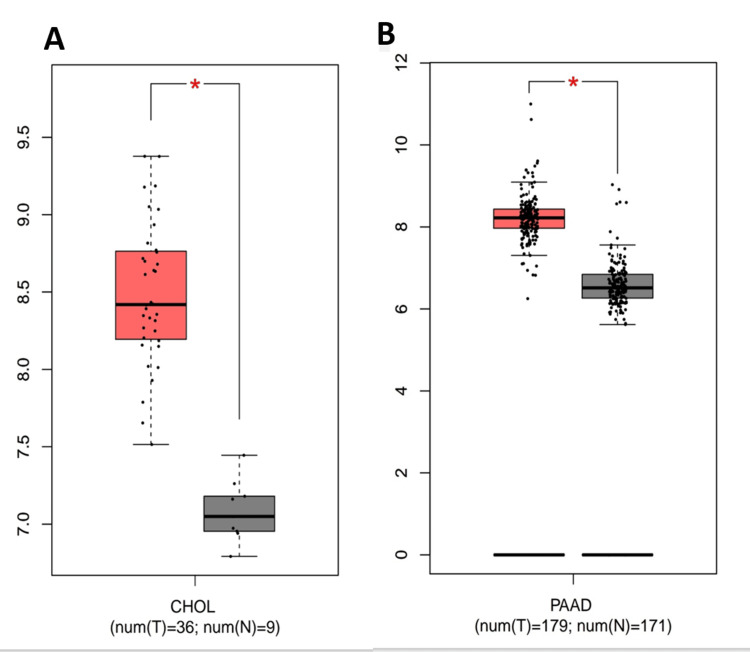
Boxplot from GEPIA showing the mRNA expression levels of RNASEK in CHOL (A) and PAAD (B) via data from the TCGA database The tumor tissues are represented by red dots and boxes, whereas the normal tissues are represented by gray dots and boxes. P value= *p ≤ 0.05, **p ≤ 0.01, ***p ≤ 0.001.

**Figure 9:**
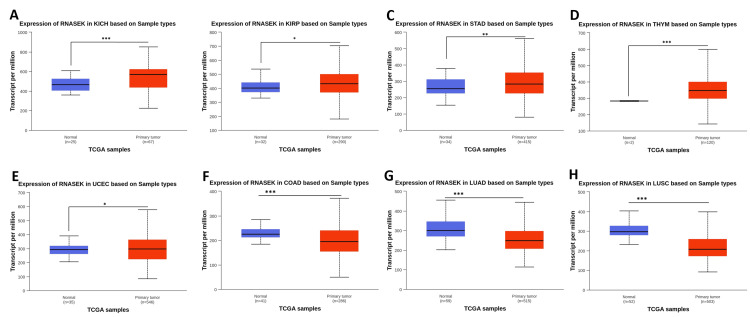
Boxplot from UALCAN databases showing the mRNA expression levels of SNHG8 gene in normal and cancer tissues via data from the TCGA database KICH (A), KIRP (B), STADP (C), THYM (D), UCEC (E), COAD (F), LUAD (G), LUAC (H). P value= *p ≤ 0.05, **p ≤ 0.01, ***p ≤ 0.001. The tumor tissues are represented by red boxes, whereas the normal tissues are represented by blue boxes

**Figure 10:**
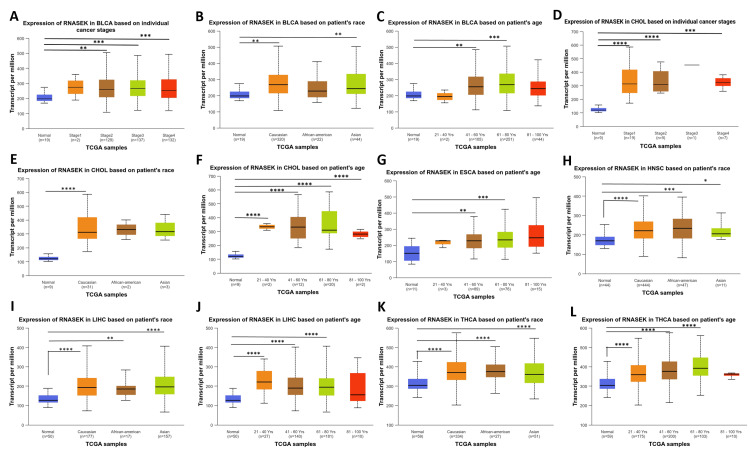
Data from UALCAN database demonstrating the mRNA expression levels of RNASEK in normal and cancer tissues among stages, race and age Boxplot demonstrating the mRNA expression levels of RNASEK in normal and cancer tissues among stages, race and age in BLCA (A, B, C), CHOL (D, E, F), age in ESCA (G), race in HNSC (H), and race and age in LIHC (I, J), and THCA (K, L) via data from the TCGA database. Normal samples are represented by blue boxes, whereas tumor samples are represented by orange boxes for stage I, brown boxes for stage II, green boxes for stage III and red boxes for stage V (p value= *p ≤ 0.05, **p ≤ 0.01, ***p ≤ 0.001).

**Figure 11:**
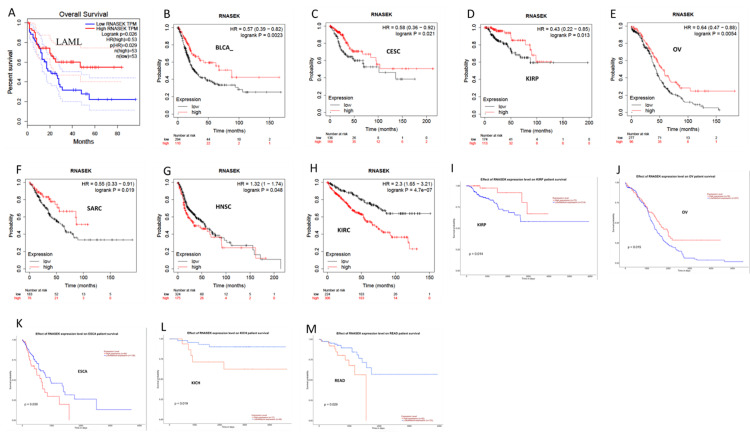
Representative survival curves from the prognostic analysis of the RNASEK survival level via a Kaplan–Meier (KM) plot in GEPIA, UALCAN and Kaplan–Meier A: LAML B: BLCA, C: CESC, D: KIRP, E: OV, F: G: HNSC, H: KIRC, I: KIRP, J: OV, K: ESCA, L: KICH, M: READ. A: Survival plot generated via GEPIA. B to H: Survival plot generated via KM plotter. Survival plot from I to M generated by UALCAN

## Appendices

Appendix A

Appendix B

Appendix C

Appendix D
